# A Rare Case of Hypothermia Due to Quetiapine Use

**DOI:** 10.7759/cureus.110654

**Published:** 2026-06-11

**Authors:** Swathi Muthyam Mogulla, Sravani Kamatam

**Affiliations:** 1 Internal Medicine, St. Dominic Hospital, Jackson, USA; 2 Internal Medicine, Order of Saint Francis (OSF) Saint Francis Medical Center, Peoria, USA

**Keywords:** antipsychotics, deprescribing, healthcare utilization, hypothermia, polypharmacy

## Abstract

Hypothermia is defined as a core body temperature of less than 35°C or 95°F. It is a rare condition with clinical manifestations ranging from asymptomatic bradycardia to death. While sepsis and endocrine abnormalities are common causes, drug-induced hypothermia is being seen lately. Antipsychotics such as risperidone and olanzapine are reported to cause hypothermia, but very few case reports exist on quetiapine-induced hypothermia. We present the case of an 80-year-old gentleman who was on quetiapine for his behavioral disturbances secondary to dementia. While he had been on the medication for years, with no new dosage adjustments, he was noted to have significant hypothermia, causing a prolonged hospital stay. After a thorough workup ruling out other causes of hypothermia, it was attributed to be a side effect of quetiapine. Hypothermia resolved within a few hours of holding the medication. This case underscores the importance of considering quetiapine-induced hypothermia in the differential diagnosis, as timely intervention may prevent prolonged hospitalization and functional decline.

## Introduction

Hypothermia is a rare and potentially fatal condition, defined as a core body temperature below 35°C/95°F. While sepsis, infections, and endocrine causes are frequently associated, medications can cause serious hypothermia [1]. Elderly patients are more vulnerable to hypothermia due to comorbidities, malnutrition, risk of sepsis, and polypharmacy [2]. General anesthetic agents, beta blockers, and antipsychotics are commonly implicated [3]. While hyperthermia and neuroleptic malignant syndrome are more commonly recognized complications of antipsychotics, hypothermia is often overlooked due to its subtle presentation. Symptoms may range from mild cardiovascular instability to coma and death. The exact pathophysiology of antipsychotic-induced hypothermia is not fully understood, but is thought to be secondary to the dopaminergic and serotonergic antagonism of these agents [4]. Treatment of hypothermia involves passive or active external and internal rewarming based on the severity.

Quetiapine-induced hypothermia is an uncommon and often overlooked adverse drug reaction. We report this case to increase awareness of quetiapine-associated hypothermia, highlight the importance of a thorough medication review in the evaluation of unexplained hypothermia, and emphasize the potential consequences of delayed recognition, including prolonged hospitalization, increased healthcare utilization, and preventable complications.

## Case presentation

An 80-year-old African-American gentleman with a history of dementia with behavioral disturbances, hypertension, hyperlipidemia, chronic kidney disease stage III, prostate cancer, and glaucoma was brought to the emergency department by his wife for concerns of confusion, generalized weakness, a fall, and poor oral intake. His wife stated that at baseline, he spoke in short sentences, but followed commands, had occasional episodes of confusion and agitation, especially in the evenings. She stated that for the past week, he refused to eat or drink and was very confused, with worsening agitation compared to his baseline.

**Figure 1:**
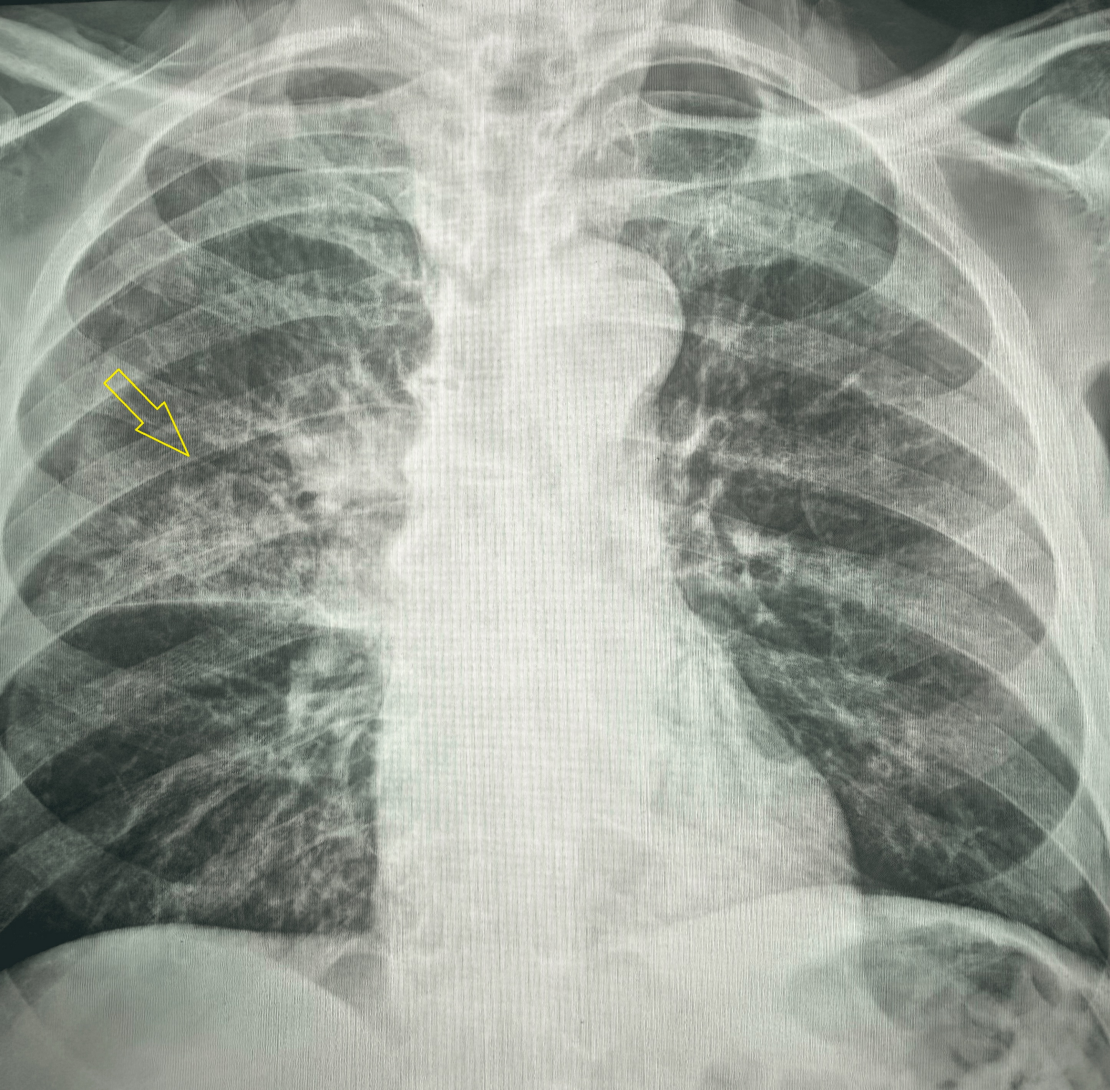
Chest X-ray findings. Chest radiograph demonstrating a right perihilar infiltrates (yellow arrow) consistent with pneumonia.

At admission, he was vitally stable, pleasantly confused, and not in distress. CT of the head showed no acute intracranial findings. Chest X-ray showed right perihilar infiltrates typical of pneumonia, as shown in Figure 1. Laboratory workup at admission is included in Table 1.

**Table 1:** Laboratory workup at admission. Laboratory findings at admission were notable for severe hypernatremia, hyperosmolality, and acute kidney injury. Endocrine evaluation, including thyroid-stimulating hormone, morning cortisol levels, and blood glucose levels, was unremarkable.

Laboratory parameter	Reference range	Value
Serum sodium	136–145 mmol/L	176
Bicarbonate	22–33 mmol/L	24
Blood urea nitrogen	5–25 mg/dL	75
Creatinine	0.73–1.18 mg/dl	3.08
Anion gap	5–13 mmol/L	12
Serum osmolality	280–300 m Osm/kg	391
Lactic acid	0.5–2.2 mmol/L	1.8
Urine osmolality	40–1,400 mOsm/kg	850
Urine sodium random	>20 mmol/L	82
Urine ketones	-	Negative
White blood cell	4–11 × 1,000µ/L	13.7
Hemoglobin	14–18 g/dL	10.3
Hematocrit	42–52%	33
Platelets	150–375 K/µL	389
Thyroid-stimulating hormone	0.35–4.94 µIU/mL	4.8
Serum AM cortisol level	4–22 µg/dL	12.6
Glucose	70–140 mg/dL	176

The patient was admitted to the intensive care unit for severe hypernatremia, and nephrology was consulted. He was initiated on an intravenous (IV) dextrose drip. Ultrasound of the kidney, ureter, and bladder showed no acute findings. A Foley catheter was placed to monitor output. He was initiated on IV ceftriaxone and azithromycin for pneumonia. His home medications, losartan and fenofibrate, were held at admission due to acute kidney injury; amlodipine, memantine, and latanoprost eye drops were continued. A speech therapist was consulted, and a modified barium swallow was done. The patient was noted to have silent aspiration with all consistencies of food and had increased fatigue over the course of the study, which increased his risk of airway compromise. The speech therapist recommended alternative means of nutrition/permanent feeding tube. Palliative care was consulted, and a goals-of-care meeting was held with the patient's wife and children, who opted for percutaneous endoscopic gastrostomy (PEG) tube placement. Gastroenterology was consulted. His serum sodium improved to 165->158->152->146->138 mmol/L, and renal function improved to baseline.

**Figure 2:**
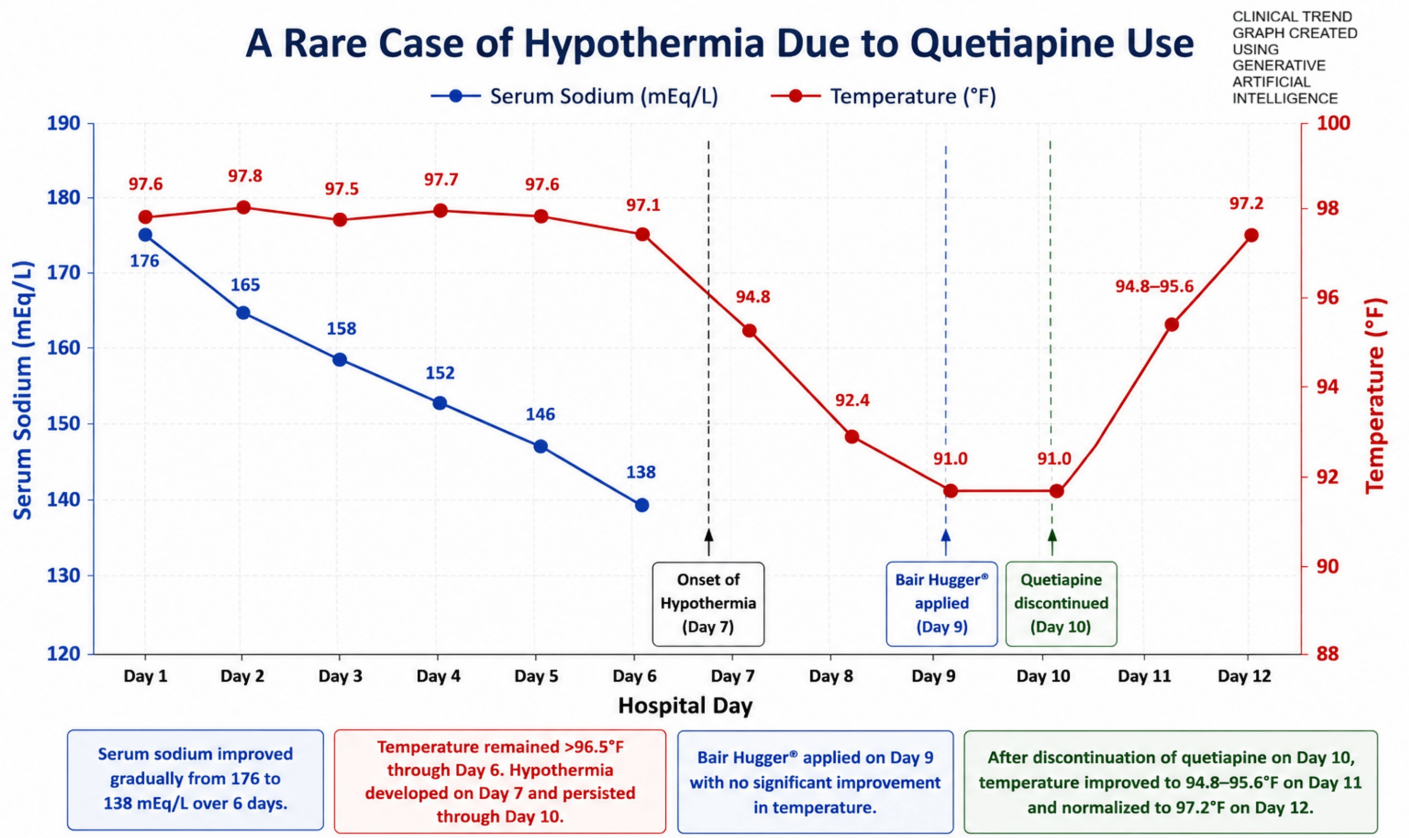
Clinical trend graph showing serum sodium levels and core body temperature over the hospital course, highlighting the onset of hypothermia and subsequent improvement following discontinuation of quetiapine. Clinical course of serum sodium levels and body temperature during hospitalization. Serum sodium levels gradually improved from 176 mEq/L on admission to 138 mEq/L by hospital day six. Despite correction of hypernatremia, the patient developed progressive hypothermia beginning on hospital day seven, reaching a nadir temperature of 91°F on hospital days nine and ten. Active warming with a Bair Hugger device was initiated on hospital day nine, and quetiapine was discontinued on hospital day ten. Body temperature subsequently improved, returning to normothermia by hospital day eleven.

The scheduled esophagogastroduodenoscopy (EGD) with PEG placement was canceled as the patient was noted to have a body temperature of 91°F. Repeat oral, axillary, and rectal temperatures were consistently low, ranging from 91°F to 93°F. His blood pressure remained stable, averaging around 110/70 mmHg; no episodes of hypotension were noted. He was noted to have bradycardia in the 40s. ECG revealed sinus bradycardia, which was attributed to hypothermia, as no other contributing factor was noted. The patient was otherwise asymptomatic, with no prior history of bradycardia. Blood glucose levels remained stable and slightly higher due to being on IV dextrose drip since admission. Serum thyroid-stimulating hormone and morning cortisol levels were noted within normal limits. IV hydration and antibiotics were continued, and active external rewarming with a Bair Hugger was initiated; however, the patient continued to have hypothermia with temperatures averaging around 91°F. On review of his medications, he was noted to be on quetiapine 100 mg, which was held as it was thought to possibly contribute to hypothermia. His wife stated that he had been on the medication for years with no recent changes in dosages or addition of new medications. Subsequently, his body temperature and heart rate improved to normal within a few hours. The clinical trend graph showing hypernatremia and core body temperature over the hospital course is depicted in Figure 2.

He was cleared by anesthesia for the procedure and eventually had the EGD with PEG placement by gastroenterology. He was initiated on tube feeds with close monitoring for refeeding syndrome. His family was educated on behavioral interventions to manage his symptoms, as he had adverse effects from antipsychotics.

## Discussion

Hypothermia is defined as a core body temperature below 35°C and is further classified by severity into mild (32-35°C/95-89.6°F), moderate (28-32°C/89.6-82.4°F), and severe (<28°C/<82.4°F ). Symptoms of mild hypothermia are heavy shivering and cold diuresis. Moderate hypothermia is characterized by reduced shivering, hyporeflexia, ataxia, and bradycardia. Severe hypothermia may cause hypotension, hypoventilation, oliguria, coma, cardiac arrest, and death. Accidental hypothermia refers to an unintentional decline in core temperature, typically resulting from environmental exposure (primary hypothermia) or impaired thermoregulation due to underlying medical conditions, metabolic or endocrine disorders, or medication effects (secondary hypothermia) [1].

In hospitalized patients, hypothermia is often underrecognized despite its significant clinical implications. It can exacerbate underlying disease severity and is associated with increased morbidity, mortality, prolonged hospital stays, and greater healthcare resource utilization. The pathophysiology of hospital-acquired hypothermia is multifactorial, involving dysregulation of thermogenesis, impaired vasoconstrictive responses, altered metabolic activity, and effects on coagulation and cardiovascular function. Elderly and medically complex patients are particularly vulnerable due to comorbidities such as endocrine dysfunction, sepsis, neurologic impairment, malnutrition, and polypharmacy, all of which compromise thermoregulatory capacity [2].

Medications are among the most common contributors to hypothermia in hospitalized patients. Drug-induced hypothermia has been associated with substantial mortality, particularly in moderate-to-severe cases. Common implicated agents include general anesthetics, beta-blockers, and neuroleptics (antipsychotics). Among neuroleptics, second-generation (atypical) antipsychotics are increasingly recognized as potential causes. Agents such as risperidone and olanzapine are more frequently reported; however, hypothermia associated with quetiapine (Seroquel) remains rare, with only a limited number of case reports in the literature [3]. Other side effects predominantly associated with first-generation antipsychotics are extrapyramidal symptoms, anticholinergic symptoms, and hyperprolactinemia. All antipsychotics are known to cause sedation, hypotension, seizures, prolonged QT interval, weight gain, dyslipidemia, and type 2 diabetes mellitus.

The proposed mechanisms underlying antipsychotic-induced hypothermia involve central and peripheral thermoregulatory pathways. Normally, dopamine-2 (D2) receptors help maintain the core body temperature at a constant level, and D1 is believed to be involved in modulation. Hence, D2 receptor blockage causes hyperthermia, as seen in neuroleptic malignant syndrome. D1 receptor blockade decreases body temperature. Similarly, hydroxy-tryptamine/serotonin (5-HT-1) receptor stimulation causes hypothermia, and 5-HT-2 stimulation causes hyperthermia. Atypical antipsychotics are hypothesized to act as antagonists at 5-HT-2 receptors, causing hypothermia, or agonists at 5-HT-1 receptors. Additionally, inhibition of peripheral vasoconstriction and shivering mediated through alpha-adrenergic receptor blockade impairs the body’s ability to respond to cold stress. The risk appears to be highest within the first week of initiation or dose escalation, particularly in elderly patients with multiple comorbidities [4].

In this case, the patient had several predisposing risk factors, including advanced age, dementia with behavioral disturbances, and chronic kidney disease. Although the initial presentation was related to hypernatremia, the subsequent development of hypothermia during hospitalization was clinically significant and associated with bradycardia. An extensive evaluation for other causes of hypothermia was unrevealing, with no cold exposure. Moreover, none of the common causes were identified, such as sepsis, hypoglycemia, or endocrine deficiency. Notably, the patient demonstrated improvement in both body temperature and heart rate following discontinuation of quetiapine, strongly suggesting a causal relationship. On the Naranjo Adverse Drug Reaction Probability Scale, this reaction scored a 7, which is categorized as probable adverse drug reaction (score: 5 to 8), which means the reaction followed a reasonable temporal sequence after a drug, followed a recognized response to the suspected drug, was confirmed by withdrawal but not by exposure to the drug, and could not be reasonably explained by the known characteristics of the patient’s clinical state [5]. Naranjo scoring for this patient is shown in Table 2.

**Table 2:** Naranjo Adverse Drug Reaction Probability Scale assessment for quetiapine-induced hypothermia. Application of the Naranjo Adverse Drug Reaction Probability Scale yielded a total score of 7, indicating a probable causal relationship between quetiapine use and the development of hypothermia in this patient.

Question	Response	Score
1. Are there previous conclusive reports on this reaction?	Yes	+1
2. Did the adverse event appear after the suspected drug was administered?	Yes	+2
3. Did the adverse event improve when the drug was discontinued, or a specific antagonist was administered?	Yes	+1
4. Did the adverse event reappear when the drug was readministered?	Do not know	0
5. Are there alternative causes that could, on their own, have caused the reaction?	No	+2
6. Did the reaction reappear when a placebo was given?	Do not know	0
7. Was the drug detected in blood or other fluids in concentrations known to be toxic?	Do not know	0
8. Was the reaction more severe when the dose was increased or less severe when the dose was decreased?	Do not know	0
9. Did the patient have a similar reaction to the same or similar drugs in any previous exposure?	Do not know	0
10. Was the adverse event confirmed by objective evidence?	Yes	+1
Total Naranjo Score		7
Interpretation: Probable Adverse Drug Reaction		

Treatment of hypothermia involves gradual rewarming under close monitoring of vitals. Mild hypothermia is treated with passive external rewarming (blankets), moderate hypothermia with active external rewarming (warm blankets), and severe hypothermia with warm IV fluids in the intensive care unit. Hypotension associated with antipsychotics is treated with IV hydration as well as inotropes such as dopamine, whereas bradycardia improves with rewarming. Hypothermia tends to resolve within 24-48 hours of treatment. In this case, passive external rewarming in conjunction with discontinuation of the medication resolved the hypothermia and bradycardia.

A review of the PubMed-indexed literature identified only four previously reported case reports, highlighting the rarity of this clinical entity. Schattner and Dubin reported a case of an 89-year-old lady with dementia who presented with altered mental status and hypothermia with a rectal temperature of 28.5°C/83.3°F. Infectious workup was negative, and hypothermia resolved with holding quetiapine and rewarming (passive and active) [3]. Lannemyr et al. reported a case of a 40-year-old woman who presented with altered mentation that was attributed to intoxication. She had hypotension, bradycardia, hypothermia, and required extracorporeal circulatory support. She was on 900 mg quetiapine for schizophrenia. Workup revealed a false-positive drug screening for tricyclic antidepressants, which can reportedly occur due to quetiapine [6]. Petterson et al. reported a case of a 51-year-old lady who was brought to the emergency department following intentional overdose of alcohol, 5,800 mg of quetiapine, and 240 mg of citalopram. She was hypotensive, bradycardic, and hypothermic with a temperature of 24°C/75.2°F. She required intubation and active and passive rewarming measures. The patient recovered well, and hypothermia was attributed to a combination of alcohol and medication overdose [7]. Ajayi et al. reported a case of recurrent hypothermia in a 76-year-old lady with refractory mania who was on olanzapine, quetiapine, valproate, and oxcarbazepine. These medications were up and down-titrated due to her recurrent manic symptoms, and the patient was noted to have recurrent hypothermia (with the lowest temperature of 90°F) with up-titration of antipsychotics and valproate. The patient was noted to be on 300 mg and 600 mg during these hypothermia episodes [8].

Malnutrition may have contributed to the development of hypothermia in this patient and cannot be completely excluded as a potential confounding factor. Elderly individuals with poor nutritional status are particularly susceptible to impaired thermoregulation and hypothermia [9]. However, the temporal relationship between quetiapine exposure, the onset of hypothermia, and the subsequent improvement following drug discontinuation supports quetiapine as the most likely precipitating factor. Nevertheless, the contribution of underlying malnutrition should be recognized as a limitation of this case.

## Conclusions

Elderly patients are particularly vulnerable to hypothermia due to the presence of multiple predisposing factors, including comorbid medical conditions, impaired thermoregulation, polypharmacy, and acute illness. This case highlights the importance of a comprehensive diagnostic evaluation to exclude infectious, endocrine, and metabolic causes of hypothermia while carefully reviewing medications as potential contributors. Recognition of quetiapine-induced hypothermia can be challenging, especially when other plausible etiologies coexist. Failure to identify medication-induced hypothermia may lead to delayed diagnosis, prolonged hospitalization, unnecessary diagnostic testing, increased healthcare resource utilization, and potentially serious complications. Clinicians should maintain a high index of suspicion for drug-induced hypothermia in vulnerable populations, as prompt recognition and discontinuation of the offending agent can facilitate recovery and improve clinical outcomes. Further studies are needed to better characterize the association between hypothermia and various classes of antipsychotic medications, including the impact of dosage, duration of therapy, patient-specific risk factors, and underlying comorbidities.
